# Efgartigimod combined with steroids as a fast-acting therapy for anti-SRP immune-mediated necrotizing myopathy

**DOI:** 10.3389/fneur.2025.1560483

**Published:** 2025-05-21

**Authors:** Qiqi Peng, Xiaoping Yao, Sheng Chen, Xiaofeng Li, Feifei Lin, Zhangyu Zou

**Affiliations:** Department of Neurology, Fujian Medical University Union Hospital, Fuzhou, China

**Keywords:** anti-SRP, IMNM, treatment, efgartigimod, FcRn inhibitor

## Abstract

**Background:**

Immune-mediated necrotizing myopathy (IMNM) is a rare autoimmune disease. Efgartigimod is a human IgG antibody Fc fragment, can enhance the degradation of IgG and thus may be a promising therapeutic agent for IMNM.

**Methods:**

All three patients exhibited proximal muscle weakness and markedly increased creatinine kinase (CK) levels. Moreover, the myositis antibody profile revealed positive anti-signal recognition particle (SRP) antibodies in all of them. Muscle biopsy performed on the first patient confirmed IMNM. Eventually, all three patients were diagnosed with anti-SRP IMNM. The first patient initially received methylprednisolone via intravenous injection and then commenced efgartigimod therapy. The second and third patients were treated with a combination of methylprednisolone intravenous injection and efgartigimod. The efficacy of efgartigimod treatment was evaluated by observing the changes in CK values and the manual muscle testing (MMT) score. Safety assessments included adverse events and serious adverse events.

**Results:**

Following one cycle of efgartigimod treatment (administered at a dosage of 10 mg/kg, once a week for a total of 4 injections in one period) in these three patients, CK values decreased by 79.30, 95.80, and 93.62%, respectively. Their MMT score increased from 232, 156, and 169 to 242, 258, and 229, respectively. Evidently, these three patients demonstrated significant improvement in muscle strength and decrease of serum CK levels after efgartigimod treatment. No serious adverse events were observed during the efgartigimod treatment.

**Discussion:**

Early application of efgartigimod in combination with methylprednisolone for the treatment of anti-SRP-IMNM led to a substantial decrease in CK values and effectively improved muscle strength. Consequently, Efgartigimod may prove to be an effective and safe therapeutic option for IMNM.

## Introduction

Immune-mediated necrotizing myopathy (IMNM) was initially described by the European Neuromuscular Centre (ENMC) in 2004 as a set of subtypes within idiopathic inflammatory myopathies (IIMs). Pathologically, it is characterized by necrosis of myocytes with no or little inflammatory cell infiltration (1). Subsequently, the 224th ENMC International Workshop reached a consensus regarding the definition, clinical diagnostic, and pathologic diagnostic criteria for IMNM (2). IMNM has been classified into three serological subtypes: anti-signal recognition particle (SRP) myopathy, anti-3-hydroxy 3-methylglutaryl coenzyme A reductase (HMGCR) myopathy, and seronegative myopathy. The immune-mediated mechanism underlying IMNM remains elusive. Related studies have demonstrated that anti-SRP and anti-HMGCR auto-antibodies are linked to the pathogenicity of IMNM, and there exists a significant correlation between muscle strength, creatine kinase (CK) levels, and the levels of anti-SRP antibody as well as anti-HMGCR antibodies (3–5).

The principal clinical manifestations of IMNM are symmetric muscle weakness in the proximal extremities and elevated serum creatine kinase levels. The key pathological features of IMNM are as follows: (1) the presence of necrotic muscle fibers distributed in a scattered manner; (2) the necrosis, phagocytosis, and regeneration of myocytes at different stages; and (3) inflammation mainly involving macrophages, with a relatively low degree of lymphocytic infiltration. The typical finding of electromyography (EMG) is myogenic damage.

Immune-mediated necrotizing myopathy represents a serious and therapeutically challenging disease. Owing to the lack of randomized controlled trials and large sample cohorts, the current treatment modalities are predominantly empirical. In the 224th ENMC Expert Consensus, corticosteroids, intravenous immunoglobulin (IVIG), and immunosuppressants were recommended as the principal treatment options, along with the incorporation of rituximab (RTX) therapy. In autoantibody-mediated neurological diseases, in principle, the available treatments can be categorized into two strategies: those aimed at suppressing pathogenic antibody production and those directed at eliminating pathogenic antibodies. Therapies targeting pathogenic antibody generation include CD19 and CD20 antibodies, as well as IL-6 inhibitors, while therapies for removing pathogenic antibodies include plasma exchange, IVIG, and FcRn antagonists (6).

Efgartigimod, which is an Fc fragment of human IgG1, augments its affinity for FcRn, thereby diminishing IgG recirculation and augmenting IgG degradation. Based on the ADAPT study (7), it has been authorized for the treatment of myasthenia gravis (MG) in the United States, Europe, Japan and China. There have been a series of case reports documenting the use of efgartigimod in Guillain–Barré syndrome (GBS), Neuromyelitis Optica Spectrum Disorder (NMOSD), anti-leucine-rich, glioma inactivated 1 (LGI1) antibody-associated autoimmune encephalitis (AE) and other disorders (8–11). Very recently, an observational cohort study involving 7 patients demonstrated the effectiveness of efgartigimod in the treatment of refractory IMNM (12), implying that efgartigimod has the potential to emerge as a novel treatment approach for IMNM. Herein, we present the efgartigimod treatment experience in three patients with a primary diagnosis of anti-SRP-IMNM.

## Case report

### Patient 1

A 63-year-old male patient presented with progressive bilateral lower extremity weakness accompanied by dysphagia that had persisted for 2 months. The initial symptoms were difficulty in climbing stairs and getting up from a squatting position, along with pain in both legs. Subsequently, the symptoms gradually worsen, with the emergence of dysphagia and occasionally choking coughs. Five days prior to admission, laboratory tests revealed that the serum CK level was 3,337 U/L, the CK-MB level was 249.8 U/L, and the lactate dehydrogenase (LDH) level was 686.6 U/L.

**Figure 1:**
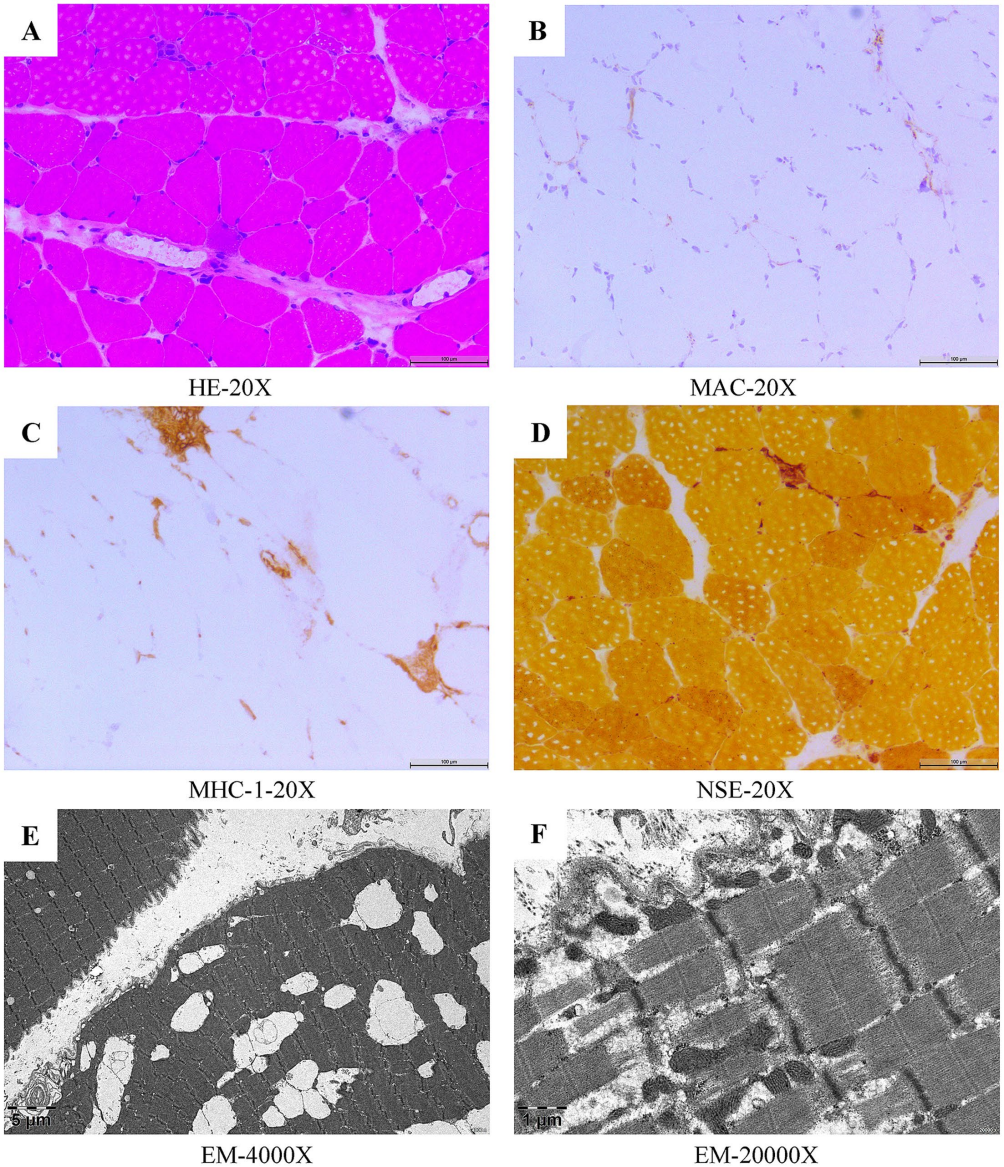
Myopathological features of the right quadriceps muscle biopsy in patient 1. Histological and immunohistochemical analysis (H&E) revealed infiltration of mononuclear phagocytes and a few lymphocytes in the fascicles and interstitial spaces of the myofibers, and scattered small angular, striated atrophic muscle fibers, and necrotic, regenerating muscle fibers, with regenerating muscle fibers predominating **(A)**. Deposits of MAC were seen on individual muscle fiber membranes **(B)**. MHC-1 expressed on individual muscle fibers **(C)**. Non-specific esterase staining (NSE) revealed a small amount of deep staining within the fasciculus, in the interstitial spaces of the muscle fibers, and scattered small amounts of deep staining of the muscle fibers **(D)**. Electron microscopic (EM) ultrastructural changes showed focal disorganization of some myofibrils, loss of focal myofibrils and mild tearing and lysis of myofibrils, occasional myocyte atrophy, mild puckering of myofibrils, myocyte size heterogeneity, mild widening of local interstitial spaces, and scattered infiltration of individual macrophages **(E,F)**.

Upon admission, a neurological examination revealed that muscle strength in the lower limbs was decreased (MRC 4/5 in distal limbs and MRC 3/5 in proximal limbs). Blood biochemical analysis demonstrated elevated levels of serum CK (2,449 U/L), CK-MB (238.5 U/L), LDH (668 U/L), AST (123 U/L), and ALT (112 U/L). Serum paraneoplastic antibody profile (Including Amphiphysin, CV2, Hu, Yo, Ma2, Ri antibodies) revealed no abnormalities. Tests of the myositis antibody profile demonstrated that both the anti-SRP antibody and the anti-SSA/Ro52kD antibody were positive. A computed tomography (CT) scan of the lung demonstrated scattered inflammation in both lungs and nodules in both lungs (consider low-risk nodules). An abdominal ultrasound revealed prostatic hyperplasia with calcification. Subsequently, a muscle biopsy of the right quadriceps muscle was conducted. Pathologic changes observed in the muscle biopsy were consistent with IMNM (Figure 1). Based on the new criteria of the ENMC, the patient was ultimately diagnosed with anti-SRP-IMNM. We selected a total of 14 muscles for the manual muscle testing (MMT) score, with all but the neck muscles divided into left and right sides, resulting in a total score of 260 (Supplementary Table 1). The initial MMT score was 232.

The patient initially received intravenous methylprednisolone pulse therapy (IVMP). The dosage was 500 mg/day for 3 consecutive days, followed by 240 mg/day for 3 consecutive days, 120 mg/day for 3 consecutive days and 80 mg/day for 3 consecutive days. After that, the patient was switched to oral prednisone at a dose of 50 mg/day, with the dosage being gradually tapered. After treatment with IVMP, the patient’s CK level gradually decreased from 2,729 U/L to 1,117 U/L. The patient’s dysphagia and bilateral lower limb weakness improved slightly, but the change in the MMT score was not significant. Subsequently, oral mycophenolate mofetil (MMF) was added to the treatment. However, even with the use of hepatoprotective drugs (glutathione combined with polyene phosphatidylcholine), the patient’s aminotransferase values remained elevated (ALT 197 U/L, AST 175 U/L). In addition, the patient had a concurrent lung infection during the hospitalization characterized by coughing, sputum production, and an elevated white blood cell count, and the albumin value gradually decreased. Since immunosuppressive drugs may further worsen liver and kidney function, and rituximab treatment may aggravate lung infections, and IVIg was not available due to a supply shortage, the treatment with MMF was discontinued. After effective anti-infection treatment of lung infection, the patient was treated with a cycle of efgartigimod at a dose of 400 mg/day, once a week for 4 weeks. During the treatment with efgartigimod, the patient was treated combined with oral prednisone (initial dose of 50 mg/day, reduced by 5 mg every 2 weeks). After one cycle of efgartigimod treatment, the patient’s CK level decreased to 230.9 U/L, the total MMT score increased to 242. The patient’s dysphagia and lower limb weakness improved compared to before, and the patient was even able to climb 7 flights of stairs. And no relevant adverse events were observed during the efgartigimod treatment. Eventually, the patient continued to be treated with oral prednisone at a dose of 20 mg/day for maintenance.

### Patient 2

An 81-year-old male patient presented with progressive limb weakness lasting for more than 1 month. Initially, the patient began to feel tenderness in the lower limbs after walking just tens of meters, and felt discomfort when lifting the upper limbs for an extended period. Five days prior to admission, the patient developed difficulties in going upstairs and lifting upper limbs. By the time of hospital admission, he was unable to walk. The patient had undergone a cholecystectomy 7 years ago. One year ago, he was diagnosed with an invasive high-grade uroepithelial carcinoma of the left ureter and subsequently had radical left nephrectomy, total excision of the left ureter, partial cystectomy, followed by postoperative pirarubicin chemotherapy.

Upon admission, a neurological examination revealed that muscle strength in the cervical flexor muscles was MRC 3/5. In the upper limbs, muscle strength was decreased (MRC 5^−^/5 in distal limbs and MRC 4/5 in proximal limbs), and in the lower limbs, it was also decreased (MRC 4/5 in distal limbs and MRC 3/5 in proximal limbs). The superficial and deep sensations of the limbs were normal. Bilateral Babinski signs were negative. Laboratory tests demonstrated that the CK level was 7,851 U/L, the CK-MB level was 229.2 U/L, the LDH level was 1,100 U/L, the ALT level was 168 U/L, the AST level was 192.7 U/L, and myoglobin was >3,000 ng/mL. Tests of the myositis antibody profile revealed anti-SRP antibody was positive. The MMT score were 156. Based on the ENMC diagnostic criteria, the patient was diagnosed with anti-SRP-IMNM.

Given that this patient had a rapidly progressive disease course, along with a previous history of left ureteral cancer and post-operative chemotherapy, a combination of efgartigimod and IVMP was used. After obtaining informed consent, the patient was treated with a cycle of efgartigimod (800 mg/day, once a week for 4 weeks), in combination with intravenous methylprednisolone treatment (240 mg/day for 3 consecutive days, 120 mg for 3 consecutive days, 80 mg for 3 consecutive days), followed by oral prednisone (50 mg/day), with the dosage gradually being tapered.

**Figure 2:**
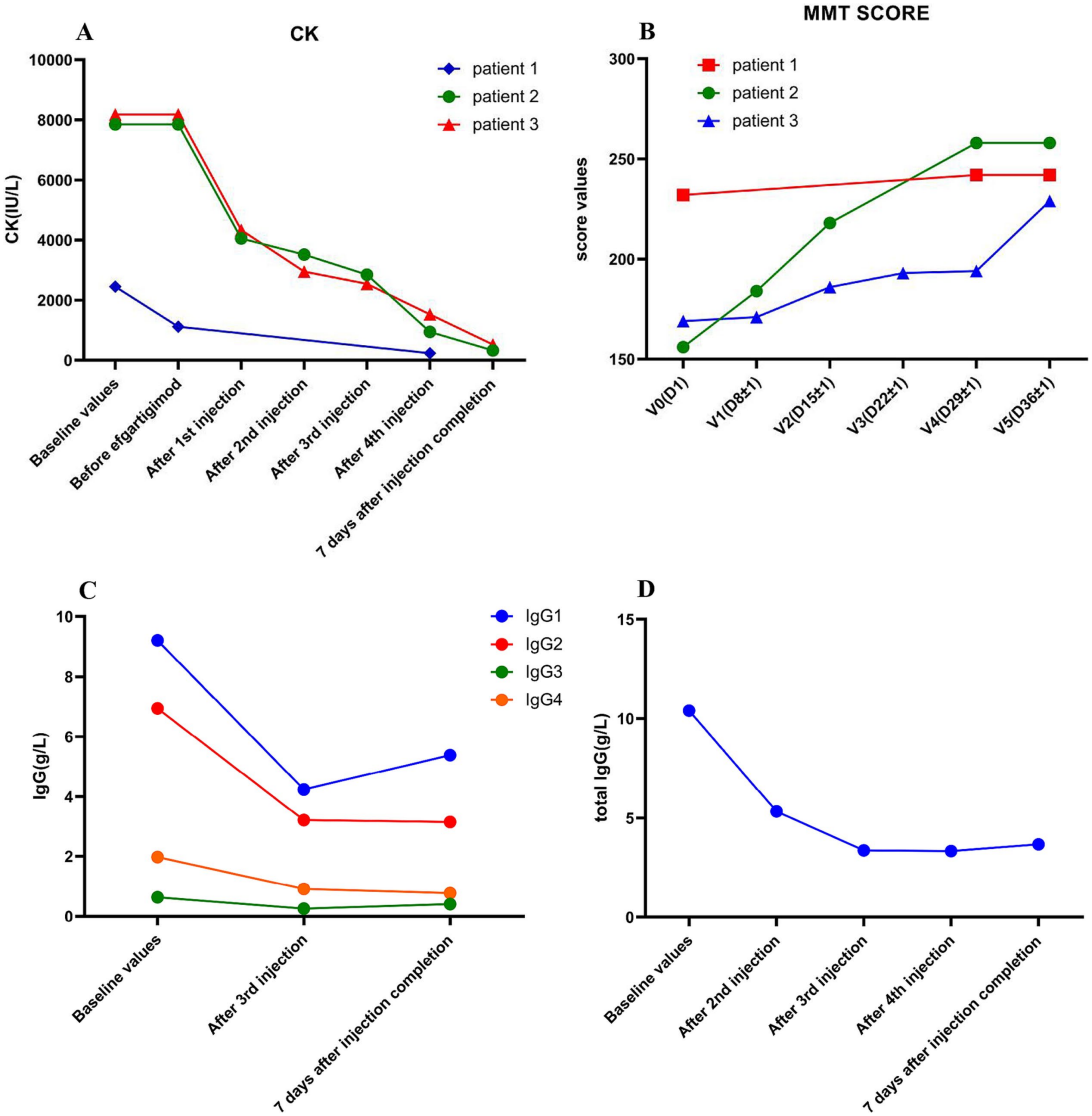
The variations of CK levels, MMT score, and serum IgG levels in the three patients before and after efgartigimod treatment. Significant decrease in CK level during efgartigimod treatment in three patients **(A)**. The MMT score significantly Increased during efgartigimod treatment in three patients **(B)**. The serum IgG1, IgG2, IgG3 and IgG4 significant decreased during efgartigimod treatment in the second patient **(C)**. The total serum IgG level showed a significant decrease during efgartigimod treatment in the third patient **(D)**.

After the first injection of efgartigimod, the serum CK level decreased from 7,851 U/L to 4,055 U/L, and the MMT score increased from 156 to 184. After the second injection of efgartigimod, muscle strength improved greatly, and the MMT score increased to 218 while the CK level decreased to 3,519 U/L. After the third injection of efgartigimod, the serum levels of IgG1, IgG2, IgG3, and IgG4 decreased from 9.21 g/L, 6.94 g/L, 0.632 g/L, and 1.970 g/L to 4.23 g/L, 3.21 g/L, 0.258 g/L, and 0.911 g/L, respectively. Seven days after the fourth injection of efgartigimod, the MMT score increased to 258 while the CK level decreased to 328 U/L (Figure 2). This patient had no adverse events throughout the treatment.

### Patient 3

A 59-year-old female patient presented with soreness and weakness in both legs for more than 5 months and weakness of both arms for more than 1 month. Initially, the patient had symmetrical soreness and swelling of both lower limbs, accompanied by a feeling of heaviness after walking. Her symptoms gradually worsened, and 2 weeks ago she developed difficulty in walking and an inability to climb the stairs. Additionally, she had weakness in both upper limbs, an inability to lift her arms above the shoulders, and difficulty in lifting his head. She was diagnosed with breast cancer 8 years ago and had a radical mastectomy, postoperative radiotherapy, and took oral exemestane for 6 years.

On admission, a neurological examination demonstrated the muscle strength of the cervical flexor muscles were MRC 2/5. Muscle strength was decreased in the upper limbs (MRC 4/5 in distal limbs and MRC 3/5 in proximal limbs) and in the lower limbs (MRC 4/5 in distal limbs and MRC 3/5 in proximal limbs). The tendon reflexes of the extremities were normal, deep and superficial sensation was normal, and pathological signs were negative. Laboratory tests showed a CK level of 8,178 U/L, the CK-MB level of 421.6 U/L, the LDH level of 1,096 U/L, the ALT level of 186 U/L, the AST level of 200 U/L, a myoglobin level of 1,593 ng/mL, and an albumin level of 36.8 g/L. Tests of the myositis antibody profile tests suggested positive anti-SRP antibody. EMG indicated myogenic damage. The MMT score was 169. The patient was diagnosed with anti-SRP-IMNM, which may be related to long-term treatment of exemestane.

The patient was reviewed in the breast surgery clinic before admission and no breast cancer recurrence or tumor metastasis was detected. Therefore, exemestane was stopped. The patient was treated with one cycle of efgartigimod (800 mg/day, once a week for 4 weeks) in combination with intravenous methylprednisolone (120 mg/day for 3 consecutive days, followed by 80 mg for another 3 consecutive days), and then was switched to oral prednisone (50 mg/day), with the dosage gradually being tapered. The patient’s serum CK level dropped significantly to 4,339 U/L after the first injection of efgartigimod, and then decreased to 2,949 U/L, 2542 U/L, and 1,524 U/L after the second, third, and fourth injections of efgartigimod, respectively. The patient’s total serum IgG decreased from 10.40 g/L to 5.33 g/L, 3.37 g/L, and 3.34 g/L after the second, third, and fourth injections of efgartigimod, respectively, while the serum IgM and IgA had no significant change. One week after one cycle of efgartigimod treatment, the patient’s MMT score increase from 169 to 229 and the CK level decreased from 8,178 U/L to 522 U/L (Figure 2). No adverse events were observed during the treatment.

## Discussion

The IIMs are a rare group of autoimmune diseases that can lead to severe muscle damage, along with significant harm to other organs, and may even pose a threat to life. Starting from the discovery of anti-SRP antibodies and the subsequent analysis of clinical features and pathological characteristics of anti-SRP polymyositis (13–15), IMNM was proposed as a new subgroup of IIMs at the 119th EMNC, based on pathological criteria. Subsequently, researchers further found that patients with anti-HMGCR had similar clinical manifestations to those with IMNM and were significantly associated with statin exposure (16, 17). At the 224th EMNC (2), the definition and diagnostic criteria of IMNM were revised to classify IMNM into three groups: (1) anti-SRP-IMNM; (2) anti-HMGCR-IMNM; and (3) seronegative IMNM. Some studies have indicated that anti-SRP-IMNM accounts for 5–15% of IIMs (18, 19), while anti-HMGCR-IMNM makes up 6–10% of IIMs (3, 17, 19). Seronegative IMNM was detected later and accounts for 10–12% of IMNM (16, 20), although some research has shown that seronegative IMNM can account for up to 1/3 of IMNM (19).

For antibody-positive IMNM, the diagnostic requirements include: elevated serum CK levels; proximal muscle weakness; and the presence of anti-SRP antibodies or anti-HMGCR autoantibodies. Muscle biopsy, electromyography, and muscle MRI are not required for diagnosing of IMNM. For antibody-negative IMNM, diagnostic conditions include: elevated serum CK levels; proximal muscle weakness; an absence of myositis-specific autoantibodies; and a muscle biopsy that meets diagnostic criteria for IMNM. In the case of antibody-negative IMNM, a muscle biopsy is mandatory (2).

All three patients we reported exhibited proximal muscle weakness, had markedly elevated CK levels, and tested positive for anti-SRP antibodies. Therefore, a diagnosis of anti-SRP-IMNM could be established. The first patient’s muscle biopsy (Figure 1) revealed a scattered small amount of necrotic and regenerated muscle fibers, macrophage infiltration and a small amount of lymphocyte infiltration in the interstitial space of muscle fibers, as well as Membrane Attack Complex (MAC) deposition and Major Histocompatibility Complex class I (MHC-1) expression, all of which were in line with the pathological changes of IMNM.

SRP is a ribonucleoprotein complex that guides nascent polypeptides into the endoplasmic reticulum. Anti-SRP antibodies can recognize different SRP components, yet the 54 kDa subunit (SRP54) serve as the primary target (21, 22). Previous studies showed that anti-SRP antibodies were present in 81% of patients as the IgG1 subtype and 29% as the IgG4 subtype. However, some patients can possess both IgG1 and IgG4 antibodies (4). In contrast, 100% of the anti-HMGCR antibodies were found to be the IgG1 subtype (23).

IgG1 has the ability to bind to antigens from a broad range of pathogens. It interacts with the Fcγ receptor through the Fc segment, activates the complement system, and promotes phagocytosis and antibody-dependent cell-mediated cytotoxicity. On the other hand, IgG4 plays an important role in immunomodulation, featuring low Fc receptor-binding and complement-activating capacity.

The pathogenic mechanism underlying anti-SRP and anti-HMGCR antibodies in IMNM remain unclear. Nevertheless, previous studies have found a strong correlation between the levels of anti-SRP and anti-HMGCR antibodies and disease activity (4, 5). Recently, *in vitro* studies have revealed that anti-SRP and anti-HMGCR antibodies induced muscle fiber atrophy, thereby confirming that both antibodies were associated with the pathogenesis of IMNM (24, 25). Bergua et al. (26) established an IMNM mouse model to confirm the pathogenicity of IgG from patients with anti-SRP and anti-HMGCR antibodies. Additionally, they discovered that the pathogenic mechanism involved complement. Subsequently, Julien et al. (27) found that early administration of the C5 complement inhibitor zilucoplan could prevent myopathy in a humanized mouse model of anti-HMGCR-IMNM. They further evaluated the therapeutic effect of IgG reduction by an FcRn inhibitor (efgartigimod) in a humanized mouse model of IMNM and determined that efgartigimod rapidly reduced the serum and muscle levels of total IgG, including pathogenic anti-HMGCR+ IgG antibodies (28). The above studies offer us novel perspectives regarding several therapeutic strategies for IMNM, such as targeting complement targeting and utilizing FcRn antagonists.

Owing to the lack of randomized controlled trials and large sample cohorts to confirm effective therapeutic agents for IMNM, the current treatment modalities are predominantly empirical. IMNM is traditionally treated with glucocorticoids and immunosuppressants. Additionally, IVIg and plasma exchange can also be utilized. Glucocorticoids are the basic drugs used in the treatment of IMNM, but there are no standardized criteria for hormone usage. Severe IIM with dysphagia and/or difficulty walking with intravenous glucocorticoids followed by oral administration. In severe cases, initial treatment with IVMP 0.5–1 g/day for 3–5 consecutive days followed by oral prednisone (2, 29). Because of the progressive bilateral lower extremity weakness with dysphagia in the first patient, we chose IVMP for 3 consecutive days. While the second and third patient also had significant limb weakness, so they were with a combination of intravenous methylprednisolone treatment (initial 240 mg/day in the second patient and 120 mg/day in third patient) to suppress inflammation. In the case of the first patient, treatment with IVMP was initially effective, but edema occurs, and after using mycophenolate mofetil, serum AST and ALT levels increased significantly. Consequently, mycophenolate mofetil was discontinued, and efgartigimod was used instead. The second and third patient had a history of cancer. The use of immunosuppressive drugs may increase the risk of a cancer recurrence as well as cause damage of livers and kidneys. Moreover, IVIg treatment could potentially raise the risk of thromboembolism. Hence, efgartigimod in combination with relatively low dose of methylprednisolone was employed for these two patients. The third patient had undergone oral exemestane therapy for 6 years. Exemestane is an aromatase inhibitor and serve as the primary drug for the adjuvant treatment of postmenopausal women with estrogen receptor-positive (ER+) breast cancer. Previous reports have indicated that aromatase inhibitor may result in the development of myopathy (14).

After one cycle of efgartigimod treatment in these three patients, the CK values decreased by 79.30, 95.80, and 93.62%, respectively. The second patient exhibited the most pronounced ratio of CK reduction and improvement in the MMT score (Figure 2). We observed a significant decrease in the serum IgG of the second patient after the third injection of efgartigimod. Specifically, the IgG1, IgG2, IgG3 and IgG4 levels decreased by 54.07, 53.75, 59.18 and 53.76%, respectively. For the third patient, the total serum IgG level decreased by 48.75, 67.60, and 67.88% after the second, third, and fourth injections of efgartigimod, respectively. Hence, the total serum IgG decreased by more than 50% after one cycle of efgartigimod treatment, which was consistent with previous studies (9, 10, 12). Moreover, the serum IgG1, IgG2, IgG3, and IgG4 also decreased by more than 50%. In the first case, although the CK values decreased by 79.30% from before efgartigimod treatment, they were 90.6% decreased from before IVMP treatment. The change in his MMT score was not as significant as the second case, possibly because he had relatively better muscle strength initially.

The 224th ENMC defined a complete remission as having normal muscle strength and normal CK level, whereas a partial remission was characterized by an improvement in MMT-8 > 110% and/or CK level that nevertheless remained > 2 times the normal range (2). However, there have also been study designs using an 80% reduction of CK levels as the study endpoint (30). Our three patients who were treated with efgartigimod could achieve partial remission. Our study demonstrated that the combination of efgartigimod and steroids therapy for IMNM can rapidly reduce CK levels and total serum IgG, while improving muscle strength, with relatively few complications.

Efgartigimod is a human IgG1 antibody Fc fragment. It can that increases its affinity for FcRn under suitable pH conditions, which accelerates the degradation of endogenous IgG for the removal of pathogenic IgG (31). Recently, efgartigimod has been shown to be efficacious and well tolerated in the treatment of myasthenia gravis (7, 32, 33), chronic inflammatory demyelinating polyneuropathy (34), and adult primary immune thrombocytopenia (35). There are also case reports indicating its positive efficacy and safety in Guillain–Barré syndrome, Neuromyelitis Optica Spectrum Disorder, and autoimmune encephalitis (8–11, 36). Previous studies have confirmed that efgartigimod reduces circulating IgG levels, prevents further myofibrillar necrosis and permits myofibrillar regeneration in humanized mouse models of IMNM (28). Very recently Yang et al. (12) also found that efgartigimod was effective in seven patients with IMNM (five cases with anti-HMGCR antibodies and two cases within anti-SRP antibodies) by promoting the degradation of endogenous IgG.

The report presented three patients with anti-SRP-IMNM. After efgartigimod treatment, these patients showed significant improvement in muscle strength, a marked decrease in CK level, and a significant reduction in total serum IgG, consistent with the results of previous studies (12). However, the patients in this report were newly diagnosed with anti-SRP-IMNM, with a median disease duration of 2.7 months (1–5 months), and two of them had cancer. They received efgartigimod combined with steroids after the diagnosis with anti-SRP-IMNM, without prior immunotherapies. In contrast, Yang et al.’s (12) study included patients with a longer disease duration, excluded patients with comorbid tumors, and all of those patients initially received high-dose prednisone and multiple immunotherapies. There are several strengths and novelties of our study relative to the prior study. First, our study emphasizes the importance of early treatment of IMNM, with three patients initiating treatment promptly after diagnosis (no history of prior treatment), and with significant treatment outcome. The CK levels of patients declined more significantly (decreased by 79.3–95.8%) and more quickly after efgartigimod treatment. We tried the treatment with efgartigimod in combination with intravenous methylprednisolone for the first time, and without the application of other immunotherapies, avoiding the interference of previous treatments. The time of therapy is very important. Early application of efgartigimod in combination with intravenous methylprednisolone may be critical, as irreversible fibrotic damage may be present in advanced patients. Yang et al. (12) found the patients had a poor outcome with longer durations and more chronic myopathic features by muscle biopsy. And the patients had no adverse events throughout the treatment. Fewer adverse events may occur with early application of efgartigimod for IMNM. Second, two patients had a history of tumor prior to the diagnosis of IMNM. The use of immunosuppressive drugs may increase the risk of a cancer recurrence. Therefore, we tried efgartigimod for the treatment of IMNM with combined tumors, and no adverse events were observed. Efgartigimod’s safety profile in this context is a critical finding. For the IMNM patients with tumors, efgartigimod may be a potential drug, but large randomized trials and cohort studies are required to evaluate its safety and efficacy. Our study also had some limitations. First, our sample size was small and it was a retrospective case report. The study’s primary focus was early efficacy of efgartigimod in the treatment of IMNM, but long-term follow-up and optimal treatment cycles were lacking. However, our study confirmed its potential for rapid effect and bridged the gap for early intervention of efgartigimod in the treatment of IMNM. Future studies should assess durability of response and optimal cycle numbers. Second, we applied efgartigimod in combination with intravenous methylprednisolone therapy for IMNM in the second and third patient, but not efgartigimod alone. Considering intravenous methylprednisolone can rapidly suppress inflammation and efgartigimod can ensure sustained IgG depletion, reducing immediate muscle damage risk. While the synergistic effect cannot be fully dissociated, the 95.8% CK decline (vs. 79.3% in IVMP-monotherapy-treated on the first patient) suggested efgartigimod’s incremental benefit. The findings suggested that early application of efgartigimod combined with steroids may be an effective treatment option for IMNM.

## Conclusion

Efgartigimod combined with steroids, when used for early treatment, can quickly lower CK levels and improves muscle strength in patients with anti-SRP-INMN. The findings indicate that the combination of efgartigimod and steroids may be an rapid – acting treatment for INMN.

## Data Availability

The original contributions presented in the study are included in the article/Supplementary material, further inquiries can be directed to the corresponding authors.

## References

[1] Hoogendijk JE Amato AA Lecky BR Choy EH Lundberg IE Rose MR. 119th ENMC international workshop: trial design in adult idiopathic inflammatory myopathies, with the exception of inclusion body myositis, 10–12 October 2003, Naarden, the Netherlands. Neuromuscul Disord. (2004) 14:337–45. doi: 10.1016/j.nmd.2004.02.006, PMID: 15099594

[2] Allenbach Y Mammen AL Benveniste O Stenzel W Allenbach Y Amato A. 224th ENMC international workshop. Neuromuscul Disord. (2018) 28:87–99. doi: 10.1016/j.nmd.2017.09.016 29221629

[3] Allenbach Y Drouot L Rigolet A Charuel JL Jouen F Romero NB. Anti-HMGCR autoantibodies in European patients with autoimmune necrotizing myopathies. Medicine. (2014) 93:150–7. doi: 10.1097/md.0000000000000028, PMID: 24797170 PMC4632910

[4] Benveniste O Drouot L Jouen F Charuel J-L Bloch-Queyrat C Behin A. Correlation of anti-signal recognition particle autoantibody levels with creatine kinase activity in patients with necrotizing myopathy. Arthritis Rheum. (2011) 63:1961–71. doi: 10.1002/art.30344, PMID: 21400483

[5] Werner JL Christopher-Stine L Ghazarian SR Pak KS Kus JE Daya NR. Antibody levels correlate with creatine kinase levels and strength in anti–3-hydroxy-3-methylglutaryl-coenzyme a reductase–associated autoimmune myopathy. Arthritis Rheum. (2012) 64:4087–93. doi: 10.1002/art.34673, PMID: 22933019 PMC3510338

[6] Prüss H. Autoantibodies in neurological disease. Nat Rev Immunol. (2021) 21:798–813. doi: 10.1038/s41577-021-00543-w, PMID: 33976421 PMC8111372

[7] Howard JF Bril V Vu T Karam C Perk S Margania T. Safety, efficacy, and tolerability of efgartigimod in patients with generalised myasthenia gravis (ADAPT): a multicentre, randomised, placebo-controlled, phase 3 trial. Lancet Neurol. (2021) 20:526–36. doi: 10.1016/s1474-4422(21)00159-9 34146511

[8] Huang S-Q Yuan Z-H Hong Y Jiang T Zhao H-D. Shi J-Q successful treatment with efgartigimod as an add-on therapy in acute attack of anti-AQP4 antibody-positive NMOSD: a case report. Neurol Sci. (2024) 45:5511–5. doi: 10.1007/s10072-024-07678-3, PMID: 38969961

[9] Zhang H Ma J Feng Y Ma H Liu D Pang X. Efgartigimod in the treatment of Guillain–Barré syndrome. J Neurol. (2024) 271:3506–11. doi: 10.1007/s00415-024-12321-4 38532142

[10] Zhu F Wang W-F Ma C-H Liang H. Jiang Y-Q resolution of anti-LGI1-associated autoimmune encephalitis in a patient after treatment with efgartigimod. J Neurol. (2024) 271:5911–5. doi: 10.1007/s00415-024-12556-1, PMID: 38981871

[11] Li Z Xu Q Huang J Zhu Q Yang X Zhang M. Efgartigimod as rescue treatment in acute phase of neuromyelitis optica spectrum disorder: a case report. Heliyon. (2024) 10:e30421. doi: 10.1016/j.heliyon.2024.e30421, PMID: 38720715 PMC11076956

[12] Yang M Yuan J Wang Y Hao H Zhang W Wang Z. Treatment of refractory immune-mediated necrotizing myopathy with efgartigimod. Front Immunol. (2024) 15:15. doi: 10.3389/fimmu.2024.1447182, PMID: 39502686 PMC11534618

[13] Kole R Fresco LD Keene JD Cohen PL Eisenberg RA Andrews PG. Alu RNA-protein complexes formed in vitro react with a novel lupus autoantibody. J Biol Chem. (1985) 260:11781–6. PMID: 4044579

[14] Reeves WH Nigam SK Blobel G. Human autoantibodies reactive with the signal-recognition particle. Proc Natl Acad Sci USA. (1986) 83:9507–11. doi: 10.1073/pnas.83.24.9507, PMID: 2432596 PMC387169

[15] Targoff IN Johnson AE Miller FW. Antibody to signal recognition particle in polymyositis. Arthritis Rheum. (1990) 33:1361–70. doi: 10.1002/art.1780330908, PMID: 2403400

[16] Christopher-Stine L Casciola-Rosen LA Hong G Chung T Corse AM Mammen AL. A novel autoantibody recognizing 200-kd and 100-kd proteins is associated with an immune-mediated necrotizing myopathy. Arthritis Rheum. (2010) 62:2757–66. doi: 10.1002/art.27572, PMID: 20496415 PMC3026777

[17] Mammen AL Chung T Christopher-Stine L Rosen P Rosen A Doering KR. Autoantibodies against 3-Hydroxy-3-Methylglutaryl-coenzyme a reductase in patients with statin-associated autoimmune myopathy. Arthritis Rheum. (2011) 63:713–21. doi: 10.1002/art.30156, PMID: 21360500 PMC3335400

[18] Rönnelid J Helmers SB Storfors H Grip K Rönnblom L Franck-Larsson K. Use of a commercial line blot assay as a screening test for autoantibodies in inflammatory myopathies. Autoimmun Rev. (2009) 9:58–61. doi: 10.1016/j.autrev.2009.03.005, PMID: 19285154

[19] Watanabe Y Uruha A Suzuki S Nakahara J Hamanaka K Takayama K. Clinical features and prognosis in anti-SRP and anti-HMGCR necrotising myopathy. J Neurol Neurosurg Psychiatry. (2016) 87:1038–44. doi: 10.1136/jnnp-2016-313166, PMID: 27147697

[20] Allenbach Y Keraen J Bouvier A-m Jooste V Champtiaux N Hervier B. High risk of cancer in autoimmune necrotizing myopathies: usefulness of myositis specific antibody. Brain. (2016) 139:2131–5. doi: 10.1093/brain/aww054, PMID: 27086869

[21] Römisch K Miller FW Dobberstein B. High S human autoantibodies against the 54 kDa protein of the signal recognition particle block function at multiple stages. Arthritis Res Ther. (2006) 8:R39. doi: 10.1186/ar1895, PMID: 16469117 PMC1526608

[22] Janda CY Li J Oubridge C Hernández H Robinson CV Nagai K. Recognition of a signal peptide by the signal recognition particle. Nature. (2010) 465:507–10. doi: 10.1038/nature08870, PMID: 20364120 PMC2897128

[23] Drouot L Allenbach Y Jouen F Charuel JL Martinet J Meyer A. Exploring necrotizing autoimmune myopathies with a novel immunoassay for anti-3-hydroxy-3-methyl-glutaryl-CoA reductase autoantibodies. Arthritis Res Ther. (2014) 16:R39. doi: 10.1186/ar4468, PMID: 24484965 PMC3979083

[24] Arouche-Delaperche L Allenbach Y Amelin D Preusse C Mouly V Mauhin W. Pathogenic role of anti–signal recognition protein and anti–3-Hydroxy-3-methylglutaryl-CoA reductase antibodies in necrotizing myopathies: Myofiber atrophy and impairment of muscle regeneration in necrotizing autoimmune myopathies. Ann Neurol. (2017) 81:538–48. doi: 10.1002/ana.24902, PMID: 28224701

[25] Allenbach Y Arouche-Delaperche L Preusse C Radbruch H Butler-Browne G Champtiaux N. Necrosis in anti-SRP+ and anti-HMGCR+ myopathies. Neurology. (2018) 90:90. doi: 10.1212/wnl.0000000000004923, PMID: 29330311

[26] Bergua C Chiavelli H Allenbach Y Arouche-Delaperche L Arnoult C Bourdenet G. In vivo pathogenicity of IgG from patients with anti-SRP or anti-HMGCR autoantibodies in immune-mediated necrotising myopathy. Ann Rheum Dis. (2019) 78:131–9. doi: 10.1136/annrheumdis-2018-213518, PMID: 30309969

[27] Julien S Vadysirisack D Sayegh C Ragunathan S Tang Y Briand E. Prevention of anti-HMGCR immune-mediated Necrotising myopathy by C5 complement inhibition in a humanised mouse model. Biomedicines. (2022) 10:10. doi: 10.3390/biomedicines10082036, PMID: 36009583 PMC9405589

[28] Julien S van der Woning B De Ceuninck L Briand E Jaworski T Roussel G. Efgartigimod restores muscle function in a humanized mouse model of immune-mediated necrotizing myopathy. Rheumatology. (2023) 62:4006–11. doi: 10.1093/rheumatology/kead298, PMID: 37335864

[29] Oldroyd AGS Lilleker JB Amin T Aragon O Bechman K Cuthbert V. British Society for Rheumatology guideline on management of paediatric, adolescent and adult patients with idiopathic inflammatory myopathy. Rheumatology. (2022) 61:1760–8. doi: 10.1093/rheumatology/keac115, PMID: 35355064 PMC9398208

[30] Mammen AL Amato AA Dimachkie MM Chinoy H Hussain Y Lilleker JB. Zilucoplan in immune-mediated necrotising myopathy: a phase 2, randomised, double-blind, placebo-controlled, multicentre trial. Lancet Rheumatol. (2023) 5:e67–76. doi: 10.1016/s2665-9913(23)00003-6, PMID: 36923454 PMC10009502

[31] Ulrichts P Guglietta A Dreier T van Bragt T Hanssens V Hofman E. Neonatal fc receptor antagonist efgartigimod safely and sustainably reduces IgGs in humans. J Clin Invest. (2018) 128:4372–86. doi: 10.1172/jci97911 30040076 PMC6159959

[32] Sacca F Barnett C Vu T Peric S Phillips GA Zhao S. Efgartigimod improved health-related quality of life in generalized myasthenia gravis: results from a randomized, double-blind, placebo-controlled, phase 3 study (ADAPT). J Neurol. (2023) 270:2096–105. doi: 10.1007/s00415-022-11517-w, PMID: 36598575 PMC10025199

[33] Howard JF Jr Vu T Li G Korobko D Smilowski M Liu L. Subcutaneous efgartigimod PH20 in generalized myasthenia gravis: a phase 3 randomized non-inferiority study (ADAPT-SC) and interim analyses of a long-term open-label extension study (ADAPT-SC plus). Neurotherapeutics. (2024) 21:21. doi: 10.1016/j.neurot.2024.e00378, PMID: 39227284 PMC11579873

[34] Allen JA Lin J Basta I Dysgaard T Eggers C Guptill J. Safety, tolerability, and efficacy of subcutaneous efgartigimod in patients with chronic inflammatory demyelinating polyradiculoneuropathy (ADHERE): a multicentre, randomised-withdrawal, double-blind, placebo-controlled, phase 2 trial. Lancet Neurol. (2024) 23:1013–24. doi: 10.1016/S1474-4422(24)00309-0 PMID: 39304241

[35] Broome CM McDonald V Miyakawa Y Carpenedo M Kuter DJ Al-Samkari H. Efficacy and safety of the neonatal fc receptor inhibitor efgartigimod in adults with primary immune thrombocytopenia (ADVANCE IV): a multicentre, randomised, placebo-controlled, phase 3 trial. Lancet. (2023) 402:1648–59. doi: 10.1016/s0140-6736(23)01460-5, PMID: 37778358

[36] Zhang Q Yang W Qian Y Zhang Y Zhao H Shu M. Case report: rapid symptom relief in autoimmune encephalitis with efgartigimod: a three-patient case series. Front Immunol. (2024) 15:1444288. doi: 10.3389/fimmu.2024.1444288, PMID: 39421741 PMC11484013

