# Supplementary material for: Efgartigimod combined with steroids as a fast-acting therapy for anti-SRP immune-mediated necrotizing myopathy
Source: Front Neurol. 2025 May 21;16:1560483. doi: 10.3389/fneur.2025.1560483 (PMC12133534; doi:10.3389/fneur.2025.1560483)
Supplement: Supplementary file 1 [file Table_1.DOC]

Table 1 The Manual Muscle Testing score

|  | muscle group | Score |
| --- | --- | --- |
| 1 | Neck **extensors** |  |
| 2 | **Neck flexors** |  |
| 3 | Trapezius | left |
|  |  | right |
| 4 | Deltoid | left |
|  |  | right |
| 5 | Biceps | left |
|  |  | right |
| 6 | **Wrist extensors** | left |
|  |  | right |
| 7 | Wrist **flexors** | left |
|  |  | right |
| 8 | Gluteus maximus | left |
|  |  | right |
| 9 | Gluteus medius | left |
|  |  | right |
| 10 | Iliopsoas | left |
|  |  | right |
| 11 | Biceps femoris | left |
|  |  | right |
| 12 | Quadriceps femoris | left |
|  |  | right |
| 13 | Tibialis anterior | left |
|  |  | right |
| 14 | Gastrocnemius | left |
|  |  | right |
|  | Totle points | **|＿|＿|＿|** |
